# A randomized open-labeled study to examine the effects of creatine monohydrate and combined training on jump and scoring performance in young basketball players

**DOI:** 10.1080/15502783.2022.2108683

**Published:** 2022-08-08

**Authors:** Salvador Vargas-Molina, Manuel García-Sillero, Richard B. Kreider, Enrique Salinas, Jorge L. Petro, Javier Benítez-Porres, Diego A. Bonilla

**Affiliations:** a Department of Sport Sciences, EADE-University of Wales Trinity Saint David, Málaga, Spain; b University of Málaga, Physical Education and Sport, Faculty of Medicine, Spain; c College Station, Human Clinical Research Facility, Texas A&M University, Exercise & Sport Nutrition Lab, Texas, USA; d Physical Education and Sport Area, Research Group in Physical Activity, Sports and Health Sciences, Universidad de Córdoba, Montería, Colombia; e Dynamical Business & Science Society – DBSS INTERNATIONAL, Research Division, Bogotá, Colombia; f Universidad Distrital Francisco José de Caldas, Research Group in Biochemistry and Molecular Biology, Bogotá, Colombia; g University of the Basque Country UPV/EHU, Sport Genomics Research Group, Department of Genetics, Physical Anthropology and Animal Physiology, Faculty of Science and Technology, Donostia-San Sebastián, Spain

**Keywords:** Resistance training, power, physical fitness, adolescents, dietary supplements

## Abstract

**Background:**

Creatine monohydrate (CrM) supplementation has been shown to be an effective and safe nutritional supplement to improve performance; however, the impact of CrM supplementation in young basketball players is less clear. This study evaluated the effects of CrM supplementation during a strength and conditioning training (SCT) program on lower-limb strength parameters and performance in under-16 (U16) basketball players.

**Methods:**

Twenty-three male U16 basketball players participated in this study (14.3 ± 0.4 years; BMI: 20.7 ± 2.2 kg∙m^−2^). The players were randomly assigned to either a CrM group (*n* = 12) that ingested 0.1 g·kg^−1^·day^−1^ of CrM or to a non-supplemented control group (*n* = 11, CON). The athletes participated in an 8-week SCT program consisting of two lower-limb resistance-training sessions and two plyometric sessions per week. Squat jump (SJ), drop jump (DP), countermovement jump (CMJ), and Abalakov (ABK) jump power tests as well as basketball performance (points and minutes per game) were measured before, during and/or after the intervention. Data were analyzed using a general linear model with repeated measures with independent Student’s t-test pairwise comparisons.

**Results:**

The results (95% confidence interval for mean change from baseline) show that there were significant differences for all variables for CrM and CON, respectively: SJ (cm): 2.6 – 6.4, P < 0.01 and 2.2–5.1 P < 0.01; DJ (cm): 2.5–5.6, P < 0.01, and 1.8–4.4, P < 0.01; CMJ (cm): 0.3–0.8, P < 0.01, and 0.2–0.5, P < 0.01; ABK (cm): 2.8–5.5, P < 0.01 and 0.7–2.6, P = 0.003. A significant group x time interaction (*p* = 0.003, *η_p_^2^ *= 0.342) was observed in ABK performance. No significant group x time effects were seen in squat jump (*p* = 0.449, *η_p_^2^ *= 0.028), drop jump (*p* = 0.143, *η_p_^2^ *= 0.099), or counter movement jump (*p* = 0.304, *η_p_^2^ *= 0.05). A significant interaction effect was also observed in points per game (*p* = 0.049, *η_p_^2^ *= 0.149), while a non-significant but medium effect was seen in minutes per game (*p* = 0.166, *η_p_^2^ *= 0.094).

**Conclusions:**

CrM supplementation in conjunction with resistance and plyometric training increased the lower-limb ABK power and scoring performance in U16 basketball players.

## 1. Background

Creatine (N-(aminoiminomethyl)-N-methyl glycine) is an endogenous metabolite that is derived endogenously from the amino acids arginine, glycine, and ornithine and mainly synthesized in the in the liver, kidney, and pancreas [1]. Endogenously synthesized creatine (Cr) as well as creatine obtained primarily from meat and fish in the diet circulates through the blood and then is taken up into tissues through creatine transporters [1]. Once in the cell, about two-thirds of creatine is phosphorylated by creatine kinase (CK) to produce phosphocreatine (PCr) [2] with the remaining creatine pool available to be phosphorylated [3]. Due to their low molecular weight and less charge, Cr and PCr are involved in the efficient resynthesis of adenosine triphosphate (ATP) during energy metabolism via the phosphagen or CK/PCr system [4]. The CK/PCr system is a primary component of muscle bioenergetics during high-intensity exercise as well as plays an important role as a spatio-temporal energy buffer, hydrogen ion (H^+^) buffer, and low-threshold ADP sensor [1,4,5].

One of the most widely studied nutritional ergogenic aids over the last 40 years has been creatine monohydrate (CrM) [3,6]. CrM supplementation has been shown to not only promote positive effects on exercise performance and training adaptations [6,7] but also a number of potential therapeutic benefits [3], including children and adolescents [3,6,8–10]. In team sports that typically involve high-intensity intermittent exercise, PCr is an important and readily available source of energy in the cytosol. For example, Latzel et al. [11] found that German under-16 (U16) national basketball players rely mainly on oxidative metabolism but have an important contribution of energy from the phosphagen system. In this sense, reported improvements in fatigue prevention [12], high-intensity performance [13], jump and sprint capacity [14], body composition [15,16] and post-activation potentiation [17] might support the benefits of CrM supplementation on team sports performance such as basketball [6,7,18].

Independent of population, training intervention, or supplementation protocol, CrM supplementation has been reported to increase strength and power performance in short-duration efforts [6,7,19]. For this reason, creatine use is popular among athletes participating in a variety of sports [20], including team sports such as football, soccer, basketball, and volleyball athletes [7,21], basketball players [6], and volleyball players [22], among others. However, while the safety and ergogenic value of CrM supplementation has been well established in children and adolescents with medical conditions that may benefit from creatine supplementation, less is known about the efficacy and safety in younger competitive athletes [10]. The prevalence of the use of CrM as a dietary supplement by adolescent athletes has been reported to range between 5.3% and 34.1% with higher prevalence among males (23–72%) and international adolescent athletes (25.3–60%) [10]. Although some concerns have been raised about adolescent athletes using creatine as a dietary supplement, the International Society of Sports Nutrition position stand on creatine supplementation [6] concludes that there is no scientific evidence that children and/or adolescents should not take CrM [10]. In fact, previous long-term studies have not found side effects on hepatic, renal, or muscle injury markers in young adults (24.0 ± 4.0 years) during three competitive basketball seasons [23]. Furthermore, CrM supplementation in clinical populations as well as healthy adolescents has been shown to be well tolerated [24] and enhance the performance of adolescent swimmers and soccer [10]. Currently, there is limited research in young athletes linking the consumption of creatine monohydrate with the application of a combined training program. Therefore, the aim of this study was to administer CrM to young basketball players during an 8-week strength and conditioning training program (SCT) to assess the changes in different vertical jump tests and scoring performance. We hypothesize that CrM intake will improve jump capacity without negatively affecting score performance.

## 2. Materials and methods

### 2.1 Experimental approach to the problem

**Figure 1.**
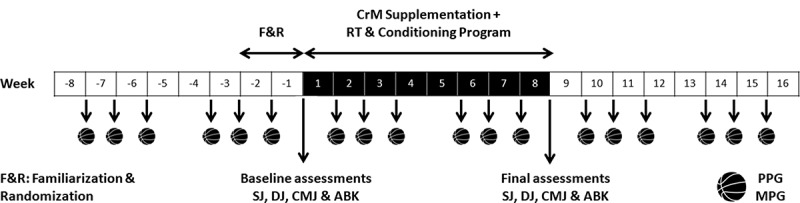
Schematic representation of the study design. ABK, Abalakov test; CMJ, countermovement jump; CrM, creatine monohydrate; DJ, drop jump; F&R, familiarization and randomization; PPG, points per game; MPG, minutes per game; RT, resistance training; SJ, squat jump.

This study was conducted as a randomized controlled study with a pretest-posttest design to assess the effects of eight weeks of CrM supplementation during a training on jumping power and basketball performance. Young players were randomly assigned to either a CrM supplemented group (CrM) or to a non-placebo control group (CON). Lower-limb power-related data (i.e. squat jump, drop jump, countermovement jump, and Abalakov jump tests) were obtained at baseline and after eight weeks of training, while sports performance was evaluated in terms of recorded points per game (PPG) and minutes per game (MPG) prior to, during and following intervention (see Figure 1).

### 2.2 Participants

The study participants were male U16 basketball players of the Unicaja Málaga reserve team, competing in the men’s Cadet Gold League of the Malaga Provincial Championship. The parents of the participants were informed of the experimental protocol and the possible associated risks. A parental informed consent was obtained for each player. Data were collected at the facilities of Unicaja Málaga and the University of Málaga. Procedures followed the Declaration of Helsinki and its later amendments and were approved by the Research Ethics Committee of the University of Málaga (code: 38-2019-H). Participants competing on the men’s U16 Unicaja Málaga reserve team basketball club were invited to participate in the study with parental consent.

### 2.3 Procedures

Four familiarization sessions were completed during the two weeks prior to the initiation of this study. Familiarization sessions were used to teach and practice plyometric movements in order to guarantee the proper execution of the technique. The last visit to the laboratory also included the baseline assessments. After this two-week period, both the CrM and CON players started the same 8-week strength and conditioning intervention program. Pretest and posttest measurements of power-related variables were performed approximately between 18:00 and 19:30 on Mondays in a neutral environment (18–20°C).

### 2.4 Anthropometry

All anthropometric data were collected during the first visit to the laboratory during the familiarization period. Body mass was measured with a digital scale to the nearest 50 grams (Tanita RD-545, Tokyo, Japan). A fixed stadiometer was used to measure the stature (SECA 220, Hamburg, Germany).

#### 2.2.1 Maximal strength assessment

In this first session, 65% of the one repetition maximum (1RM) for each exercise was calculated using Gervasport equipment (Gervasport, Madrid, Spain). Before beginning the session, participants performed a general warm-up consisting of low-intensity stationary cycling for 7–10 minutes, followed by a specific warm-up for each exercise with a range of 12–15 repetitions at 40% of the perceived 1RM. The participants then performed two to three sets of 2–3 repetitions at 60–80% of 1RM, followed by sets of one repetition until 1RM was determined. A rest interval of 3–5 minutes was provided between each attempt. The research assistants monitored the performance of the participants to ensure the correct execution of the movements using standard procedures, according to the laboratory methods described by Vargas et al. [25].

#### 2.2.2 Exercise protocol

**Figure 2.**
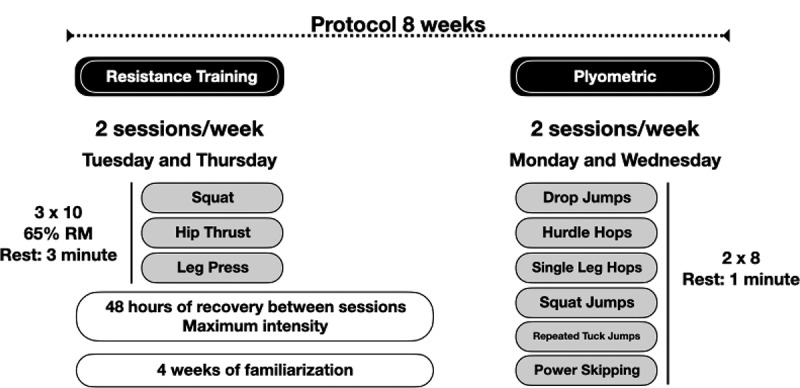
Organization of the strength training protocol.

The training program consisted of resistance-training and plyometric training. During resistance-training, two strength conditioning sessions were held per week, with 48 hours of recovery between sessions (Tuesday and Thursday). Resistance-training included, parallel (90^◦^) squats, hip thrusts, and leg press. Participants performed 3 sets of 10 repetitions 65% of 1RM, with 3-minute rest between sets, at a maximum execution speed as long as proper technique was maintained for each exercise. In addition, on the days of training with the equipment (Monday and Wednesday), 2 sets of 8 repetitions at maximum intensity of the exercises were incorporated for drop jumps, hurdle hops, single leg hops, squat jumps, repeated tuck jumps, and power skipping, with a one-minute pause between sets (see Figure 2). All training sessions were supervised by two trained and experienced sport scientists.

#### 2.2.3 Power-related variables

For the squat jump and counter movement jump, participants were instructed to start the movement squatting down to a 90^◦^ knee angle at the beginning of the jump phase and to keep their hands on their waist and their trunk upright. The instructions emphasized that the movement should be performed without interruption until completion of the jump. For the Abalakov jump test, the instructions were similar, with the difference that the arms were used to increase momentum. For the drop jump test, a 40 cm platform was used, and participants were instructed to drop from this height with their hands on their hips, to keep their trunk in full extension while in the air, and to flex their knees to an angle of 90^◦^ when they hit the ground, so that they could perform a vertical jump with the maximum impulse. The 90^◦^ knee angle was measured with a manual goniometer, and it was marked on the wall. Two researchers later confirmed the depth of the jump by observation to standardize the depth of the countermovement on the propulsive phase of the jump.

#### 2.2.4 Supplementation protocol

Basketball players in the CrM group followed a chronic creatine supplementation protocol (0.1 g·kg^−1^·day^−1^) from weeks one to eight. The CrM supplement (Creatine, Red Gold Series, MTX Corp., Irún, Spain) was fully dissolved in juice and given to the participants immediately after each training session. The compliance with CrM supplementation on non-training days was confirmed by providing daily doses to take at home with personal communication. This supplementation protocol has been shown to not only be safe but also to enable increased performance in other adolescent populations [6]. Those assigned to the CON group followed their normal dietary habits.

#### 2.2.5 Primary outcome measures

Participants were instructed to avoid vigorous exercise for the 72 hours prior to the tests and performance measures were obtained at the same time of day. Squat jump, drop jump, counter movement jump, and Abalakov jump performance was measured in centimeters by the My Jump 2® phone application (My Jump 2, Madrid, Spain), which has been previously validated [26], after a general warm-up consisting of continuous running, general calisthenics and stretching exercises. Participants performed three trials for each test with a 20-second rest interval between attempts and 10 seconds between tests based on Santos and Janeira [27]. The mean values of the three jumps were used for statistical analysis (coefficient of variation of the technician was 4.65%). Primary outcome variables were assessed prior to and following 8-weeks of mid-season supplementation.

#### 2.2.6 Secondary outcome measures

To assess basketball performance, weekly points per game (PPG) and minutes per game (MPG) were averaged during 6-weeks of an 8-week competition period prior to supplementation, during 6-weeks of the 8-week supplementation period, and during 6-weeks of the post-supplementation follow-up period (see Figure 1). This was done using the application proposed by the Andalusian Basketball Federation (Afición FAB, Córdoba, Spain) and was recorded by the referees of each game. These data are integrated in a general database, connected online and recorded immediately.

### 2.3 Statistical analyses

Participants consented to participate in this study were randomly assigned to either the CrM group (*n* = 12) or a control (CON) group (*n* = 12) using a 1:1 allocation ratio design (www.randomizer.org). Statistical analyses were performed with IBM SPSS version 25 software (IBM Corp., Armonk, NY, USA). Data were analyzed using general linear models (GLM) with repeated measures analyses. Normality and homoscedasticity were tested with the Shapiro-Wilk and the Levene tests, respectively. Sphericity assumption was verified with Mauchly’s test, multiple comparisons were made with the Bonferroni adjustment. Effect size was assessed using Partial Eta squared ($\eta_{p}^{2}$) values where 0.01 represented a small effect, 0.06 represented a medium effect, and 0.14 represented a large effect size [28]. Pairwise comparisons were made using independent Student’s t-tests with effect size (ES) calculated using Cohen’s *d*. The comparison of the change from baseline (∆ = posttest – pretest) between groups was carried out with an estimation-based method [29]. Mean changes with 95% confidence intervals (CI) completely above or below baseline were considered significantly different [30]. Data are expressed as mean ± SD or 95% CIs as noted with a significance level of 0.05 was assumed for all tests.

### 2.4 Results

**Figure 3.**
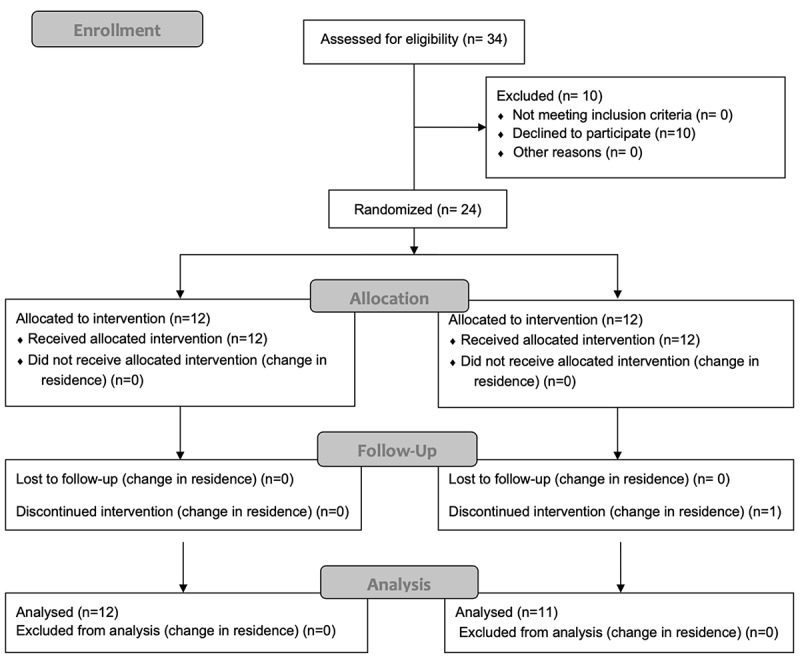
CONSORT flow diagram.

Figure 3 presents a Consolidated Standards of Reporting Trials (CONSORT) diagram. A total of 34 U16 male basketball club players from the Unicaja Málaga reserve team were invited to participate in this study. Ten declined to participate due to parental concerns about assignment to the CrM group. Twenty-four athletes and their parents consented to participate in the study. One player from the CON group discontinued the intervention due to a change in residence. A total of 32 athletes completed the study (14.3 ± 0.4 years; 66.0 ± 11.79 kg; 177.6 ± 9.0 cm; 20.7 ± 2.2 kg∙m^−2^) completed the study.

#### 2.4.1 Demographic data

Table 1 presents participant demographic data by group assignment. No significant differences were observed between groups in baseline characteristics.

**Table 1.** Characteristics of study participants.

	CrM	CON	
	*n* = 12	*n* = 11	*p*-level
Age (years)	14.3 ± 0.5	14.4 ± 0.5	0.573
Height (cm)	174.9 ± 8.3	180.5 ± 9.3	0.141
BM (kg)	62.1 ± 7.3	70.3 ± 14.5	0.097
BMI (kg m^2^)	20.3 ± 1.7	21.4 ± 2.7	0.269

Body mass (BM), Body mass index (BMI), Creatine monohydrate group (CrM), Control group (CON). Data are means ± standard deviations.

#### 2.4.2 Primary outcomes

**Figure 4.**
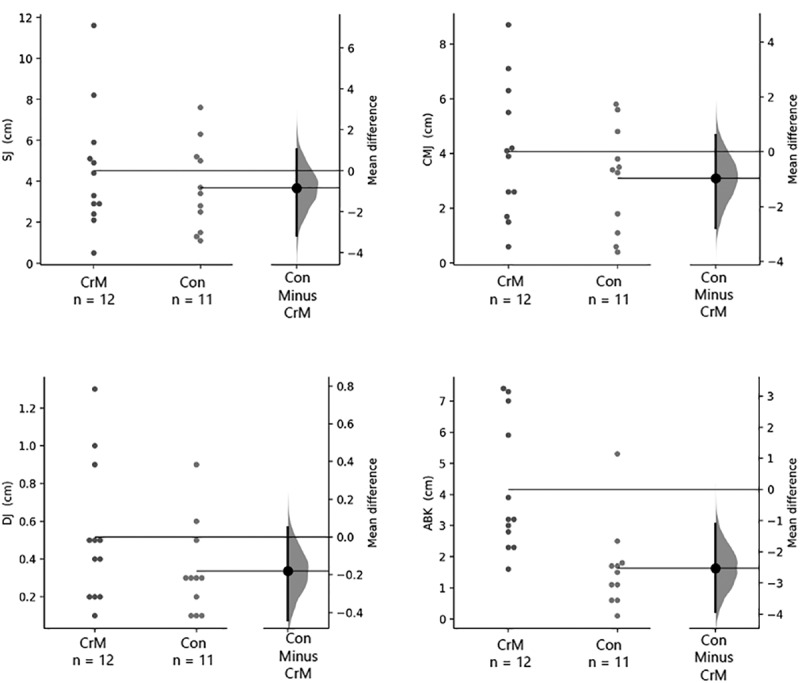
Results of the change (posttest – pretest) of the jump variables of the Creatine (CrM) and Control (Con) Groups. Both groups are plotted on the left axes; the mean difference is plotted on a floating axes on the right as a bootstrap sampling distribution. The mean difference is depicted as a dot; the 95% confidence interval is indicated by the ends of the vertical error bar. SJ, jump squat; CMJ, countermovement jump, DP, drop jump, ABK, Abalakov jump.

Significant changes (posttest – pretest) were found in all jump measurements for CrM and CON, respectively: SJ: 4.5 ± 3.0 cm (95% IC: 2.6–6.4 cm; ES: 0.9) and 3.7 ± 2.1 cm (95% IC: 2.2–5.1 cm; ES: 0.6); DJ: 4.1 ± 2.4 cm (95% IC: 2.5–5.6 cm; ES: 1.0) and 3.1 ± 1.9 cm (95% IC: 1.8–4.4 cm; ES: 0.9); CMJ: 0.5 ± 0.4 cm (95% IC: 0.3–0.8 cm; ES: 0.7) and 0.3 ± 0.2 cm (95% IC: 0.2–0.5 cm; ES: 0.4); ABK: 4.1 ± 2.1 cm (95% IC: 2.8–5.5 cm; ES: 0.6) and 1.6 ± 1.4 cm (95% IC: 0.7–2.6 cm; ES: 0.2). According to the GLM analysis, no significant group x time effects were seen in squat jump (*p* = 0.449, *η_p_^2^ *= 0.028, small effect), drop jump (*p* = 0.143, *η_p_^2^ *= 0.099, medium effect), or counter movement jump (*p* = 0.304, *η_p_^2^ *= 0.05, small-medium effect); however, significant group x time interaction (*p* = 0.003, *η_p_^2^ *= 0.342, large effect) was observed in ABK performance. Table 2 presents jump power data, while Figure 4 shows the mean changes from baseline in jump power data.

**Table 2.** Results of lower limb power-related variables.

	Group	Before	After	∆	P-value	ES	Time *p*-level (η_p_^2^)	Group *p*-level (η_p_^2^)	Time x Group *p*-level (η_p_^2^)
SJ (cm)	CrM	28.8 ± 5.3	33.3 ± 4.9	4.5 ± 3.0 (2.6, 6.4)	<0.01	0.9	<0.01 (0.727)	0.121 (0.111)	0.449 (0.028)
	CON	32.8 ± 5.3	36.5 ± 6.3	3.7 ± 2.1 (2.2, 5.1)	<0.01	0.6			
DJ (cm)	CrM	1.1 ± 0.3	1.6 ± 0.6	4.1 ± 2.4 (2.5, 5.6)	<0.01	1.0	<0.01 (0.674)	0.731 (0.006)	0.143 (0.099)
	CON	1.3 ± 0.3	1.6 ± 0.4	3.1 ± 1.9 (1.8, 4.4)	0,001	0.9			
CMJ (cm)	CrM	31.1 ± 6.1	35.2 ± 6.2	0.5 ± 0.4 (0.3, 0.8)	<0.01	0.7	<0.01 (0.742)	0.495 (0.022)	0.304 (0.050)
	CON	33.6 ± 8.1	36.7 ± 6.8	0.3 ± 0.2 (0.2, 0.5)	<0.01	0.4			
ABJ (cm)	CrM	37.0 ± 7.6	41.1 ± 7.0	4.1 ± 2.1 (2.8, 5.5)	<0.01	0.6	<0.01 (0.735)	0.282 (0.055)	0.003 (0.342)
	CON	41.7 ± 8.0	43.3 ± 7.3	1.6 ± 1.4 (0.7, 2.6)	0.003	0.2			

The results are expressed as mean ± standard deviation. ES represents Cohen’s effect size from Student´s pairwise comparisons. η_p_^2^ represents squared effect sizes where 0.01 represents a small effect, 0.06 represents medium effect, and 0.14 represents a large effect. Squat jump (SJ), Drop jump (DJ), Counter movement jump (CMJ), Abalakov jump (ABJ), Creatine monohydrate (CrM), Control (CON).

#### 2.4.3 Secondary outcomes

**Figure 5.**
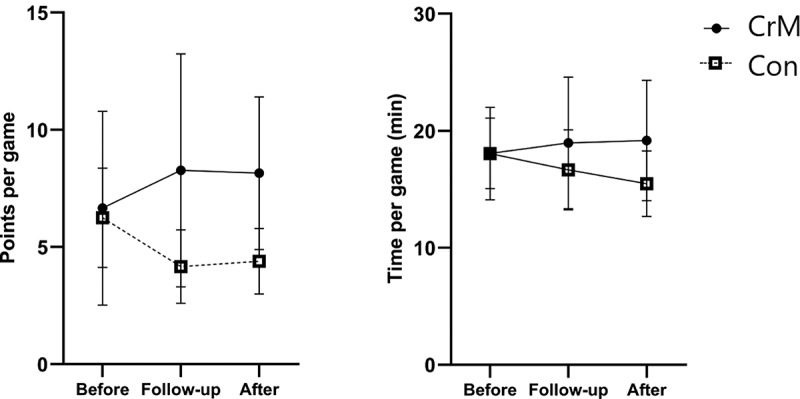
Points per game (PPG) and minutes per game (MPG) before, during and after the intervention (average of the records in 6 games in each stage). The bar indicates the 95% confidence interval.

The present study also considered the analysis of parameters related to basketball performance through the evaluation of points and minutes per game. As seen in Table 3, GLM analysis revealed a significant group x time interaction in points per game (CrM: 8.2 ± 4.9, 95% IC 4.9–11.4 and CON: 4.4 ± 2.1, 95% IC: 3.0 – 5.8; *p* = 0.049, *η_p_^2^ *= 0.149, strong effect), while a non-significant but medium effect was seen in minutes per game (*p* = 0.166, *η_p_^2^ *= 0.094, medium effect). Figure 5 presents the mean changes from baseline. Points per game significantly increased over time with CrM while decreasing in the CON group.

**Table 3.** Results of points and minutes per game.

	Before	Follow-up	After	Time P (η_p_^2^)	Group P (η_p_^2^)	Time x Group P (η_p_^2^)
**Points per game**						
CrM	6.7 ± 6.2 (2.5, 10.8)	8.3 ± 7.4 (3.3, 13.2)	8.2 ± 4.9 (4.9, 11.4)	0.985 (0.001)	0.192 (0.088)	0.049 (0.149)
CON	6.3 ± 3.0 (4.1, 8.4)	4.2 ± 2.3 (2.6, 5.7)	4.4 ± 2.1 (3.0, 5.8)			
**Minutes per game**						
CrM	18.1 ± 4.5 (15.0, 21.1)	19.0 ± 8.4 (13.3, 24.6)	19.2 ± 7.7 (14.0, 24.3)	0.613 (0.021)	0.515 (0.023)	0.166 (0.094)
CON	18.0 ± 5.5 (14.1, 22.0)	16.7 ± 5.1 (13.2, 20.1)	15.5 ± 4.2 (12.7, 18.3)			

Data shown as mean ± SD (95% confidence interval). The points and game times are the average of the record of 6 games of each phase before, during, and after the intervention (18 games in total for each group). η_p_^2^ represents squared effect sizes where 0.01 represents a small effect, 0.06 represents medium effect, and 0.14 represents a large effect. Creatine monohydrate group (CrM), Control group (CON).

## 3. Discussion

This study aimed to evaluate the effect of CrM supplementation on jump power indices as well as the game performance 16 U male basketball players. It has been shown that the application of traditional strength protocols with moderate loads over a 10 week period in 14- to 15-year-old male basketball players leads to improvements in the countermovement, squat, and Abalakov style jump performance [31]. Two days a week of plyometric exercises improve jumping ability, agility, and speed in soccer players ranging from pre-teens to 17 years of age [32]. In fact, plyometric training with an additional load has led to improvements in different vertical jump tests (i.e. CMJ and SJ), in comparison to the control group, in male basketball players [33]. Interestingly, when plyometric exercises are combined with traditional strength training, as in our study, improvements are obtained in the vertical jump, medicine ball throw and agility with regard to resistance-training only in 12 – 15 year-old boys [34]. Santos & Janeira [35] demonstrated that 14 – 15 year-old basketball players achieve significant increases in the countermovement, squat, and Abalakov jump performance with two sessions of resistance-training plus plyometric training. These results are in agreement with our findings, since an eight-week resistance and plyometric training program produced significant changes in countermovement, drop, squat, and Abalakov jump performance in both groups. However, CrM supplementation resulted in greater improvement in Abalakov-style jump performance, while moderate to large effect sizes were noted in related power variables.

Previously, Grindstaff et al. [36] provided evidence of the potential ergogenic effects of CrM administration (21 g·day^−1^ x 9 days) on upper-body performance in youth athletes in one of the first studies in this population. Izquierdo and associate [37] also reported positive effects on CMJ after an acute CrM supplementation protocol (20 g·day^−1^ x 5 days) in U16 national D-1 handball players. However, a recent study by Viera and coworkers [38] concluded that short-term CrM supplementation (20 g·day^−1^ plus 20 g·day^−1^ maltodextrin x 7 days) had no significant effects on lower-limb muscle endurance following an acute bout of 30-min exercise at 80% maximum velocity in trained young men. Rosene et al. [39] also reported that CrM supplementation did not attenuate exercise-induced muscle damage with short-term supplementation, but the ergogenic effect appeared after 30 days of supplementation. Our study demonstrated that eight weeks of CrM supplementation in conjunction with resistance and plyometric training produced a significant increase in vertical jump performance from baseline in the Abalakov power test compared to training without supplementation, while other tests had medium-to-large effect sizes suggesting those findings may be significant with a larger cohort of athletes studied. Furthermore, some studies have reported different sport-specific skills [40], such as basketball performance-related variables in U16 players, including 2-points, triples, offensive rebounds, assistances and others [41,42]. It is worth noting that CrM supplementation has been shown to reduce the fatigue index [43], which might affect precision skillsfor example, in shooting [44]. However, in terms of fatigue, it seems that more experienced athletes have a better response than less adapted individuals [45].

As a secondary outcome, our research assessed the changes in basketball performance as assessed by evaluating points and minutes per game before, during and after CrM supplementation. Our findings suggest that the intervention strategy used in this trial might benefit score performance in young basketball players, since that there were between-group differences in PPG after the intervention. Thus, this study provides evidence of the improvement in training adaptations after eight weeks of a strength and conditioning program. Since none of the subjects experienced negative side effects during the treatment, these results reinforce the recommendations of CrM supplementation for under-16 young basketball athletes involved in competitive supervised training.

## 4. Limitations

This randomized controlled trial has several flaws that should be mentioned. First, the fact that there was no placebo control group and that this was an open-label study might have influenced the findings (placebo effect). However, it is important to note that open-label trials may be appropriate for comparing two very similar treatments to determine which is most effective [46]. It should also be noted that there is a lack of consensus on standardizing assessments for the countermovement and squat in populations between the ages of 12–18 years. In the literature, discrepancies have been found in the jump technique, number of jumps and devices used. Specifically, in both male and female basketball players, jump performance varies considerably, one possible cause being differences in test protocols and player skill levels [47]. Finally, a relevant aspect when evaluating adolescents is biological age. In this regard, the differences in peak height velocity and the corresponding hormonal characteristics of each individual might alter the adaptations to strength training. This limitation might be partially controlled with the Tanner scale to determine sexual maturity.

## 5. Conclusion

CrM supplementation might benefit strength-training adaptations and sports performance in U16 basketball players undergoing a resistance and plyometric training program. Particularly, CrM supplementation increased power production in one out of four power tests and points per game scored per game in U16. No side effects were reported by the adolescents, which helps to promote safe evidence-based practices in team sports leagues with parental support for its use. CrM may therefore serve as a safe nutritional alternative to other potentially dangerous practices (e.g. anabolic androgenic steroids). Further research is needed to evaluate the influence of biological maturation.

## References

[1] Brosnan JT, Brosnan ME. Creatine: endogenous metabolite, dietary, and therapeutic supplement. Annu Rev Nutr. 2007;27:241–261. 17430086 10.1146/annurev.nutr.27.061406.093621

[2] Chapman MS. (). The structural enzymology of arginine kinase and its implications for creatine kinase. Creatine Kinase. Nova Science Publishers, Inc. 69–94 2006. 1-59454-715-7

[3] Kreider RB, Stout JR. Creatine in health and disease. Nutrients. 2021;13(2):447. 33572884 10.3390/nu13020447 PMC7910963

[4] Wallimann T, Harris R. Creatine: a miserable life without it. Amino Acids. 2016;48(8):1739–1750. 27422546 10.1007/s00726-016-2297-x

[5] Bonilla DA, Kreider RB, Stout JR, et al. Metabolic basis of creatine in health and disease: a bioinformatics-assisted review. Nutrients. 2021;13(4):1238. 33918657 10.3390/nu13041238 PMC8070484

[6] Kreider RB, Kalman DS, Antonio J, et al. International society of sports nutrition position stand: safety and efficacy of creatine supplementation in exercise, sport, and medicine. J Int Soc Sports Nutr. 2017;14:18. 28615996 10.1186/s12970-017-0173-z PMC5469049

[7] Wax B, Kerksick CM, Jagim AR, et al. Creatine for exercise and sports performance, with recovery considerations for healthy populations. Nutrients. 2021;13(6):1915. 34199588 10.3390/nu13061915 PMC8228369

[8] de Souza ESA, Pertille A, Reis Barbosa CG, et al. Effects of creatine supplementation on renal function: a systematic review and meta-analysis. J Ren Nutr. 2019;29(6):480–489. 31375416 10.1053/j.jrn.2019.05.004

[9] Rawson ES, Dolan E, and Bryan Saunders ME.2019. Creatine supplementation in sport, exercise and health Dietary Supplementation in Sport and Exercise. (Routledge). 141–164. doi:10.4324/9780429465567-7 .

[10] Jagim AR, Kerksick CM. Creatine supplementation in children and adolescents. Nutrients. 2021;13(2):664. 33670822 10.3390/nu13020664 PMC7922146

[11] Latzel R, Hoos O, Stier S, et al. Energetic profile of the basketball exercise simulation test in junior elite players. Int J Sports Physiol Perform. 2018;13(6):810–815. 29182413 10.1123/ijspp.2017-0174

[12] Dabidi Roshan V, Babaei H, Hosseinzadeh M, et al. The effect of creatine supplementation on muscle fatigue and physiological indices following intermittent swimming bouts. J Sports Med Phys Fitness. 2013;53(3):232–239. 23715246

[13] Juhasz I, Gyore I, Csende Z, et al. Creatine supplementation improves the anaerobic performance of elite junior fin swimmers. Acta Physiol Hung. 2009;96(3):325–336. 19706374 10.1556/APhysiol.96.2009.3.6

[14] Ramirez-Campillo R, Gonzalez-Jurado JA, Martinez C, et al. Effects of plyometric training and creatine supplementation on maximal-intensity exercise and endurance in female soccer players. J Sci Med Sport. 2016;19(8):682–687. 26778661 10.1016/j.jsams.2015.10.005

[15] Aguiar AF, Januario RS, Junior RP, et al. Long-term creatine supplementation improves muscular performance during resistance training in older women. Eur J Appl Physiol. 2013;113(4):987–996. 23053133 10.1007/s00421-012-2514-6

[16] Antonio J, Ciccone V. The effects of pre versus post workout supplementation of creatine monohydrate on body composition and strength. J Int Soc Sports Nutr. 2013;10:36. 23919405 10.1186/1550-2783-10-36 PMC3750511

[17] Wang CC, Yang MT, Lu KH, et al. The effects of creatine supplementation on explosive performance and optimal individual postactivation potentiation time. Nutrients. 2016;8(3):143. 26959056 10.3390/nu8030143 PMC4808872

[18] Mujika I, Burke LM. Nutrition in team sports. Ann Nutr Metab. 2010;57(2):26–35. 21346334 10.1159/000322700

[19] Lanhers C, Pereira B, Naughton G, et al. Creatine supplementation and lower limb strength performance: a systematic review and meta-analyses. Sports Med. 2015;45(9):1285–1294. 25946994 10.1007/s40279-015-0337-4

[20] Parr MK, Schmidtsdorff S, Kollmeier AS. Nutritional supplements in sports - sense, nonsense or hazard? Bundesgesundheitsblatt Gesundheitsforschung Gesundheitsschutz. 2017;60(3):314–322. 28058459 10.1007/s00103-016-2498-1

[21] Tawfik S, El Koofy N, Moawad EM. Patterns of nutrition and dietary supplements use in young Egyptian athletes: a community-based cross-sectional survey. PLoS One. 2016;11(8):e0161252. 27529492 10.1371/journal.pone.0161252 PMC4987011

[22] Amatori S, Sisti D, Perroni F, et al. Which are the nutritional supplements used by beach-volleyball athletes? A cross-sectional study at the Italian national championship. Sports (Basel). 2020;8(3). DOI:10.3390/sports8030031. PMC7183069 32168730

[23] Schroder H, Terrados N, Tramullas A. Risk assessment of the potential side effects of long-term creatine supplementation in team sport athletes. Eur J Nutr. 2005;44(4):255–261. 15309421 10.1007/s00394-004-0519-6

[24] Jagim AR, Stecker RA, Harty PS, et al. Safety of creatine supplementation in active adolescents and youth: a brief review. Front Nutr. 2018;5:115. 30547033 10.3389/fnut.2018.00115 PMC6279854

[25] Vargas-Molina S, Petro JL, Romance R, et al. Effects of a ketogenic diet on body composition and strength in trained women. J Int Soc Sport Nutr. 2020;17(1). DOI:10.1186/s12970-020-00348-7. PMC7146906 32276630

[26] Haynes T, Bishop C, Antrobus M, et al. The validity and reliability of the My Jump 2 app for measuring the reactive strength index and drop jump performance. J Sport Med Phys Fit. 2019;59(2):253–258. 10.23736/S0022-4707.18.08195-1 29589412

[27] Santos EJ, Janeira MA. The effects of plyometric training followed by detraining and reduced training periods on explosive strength in adolescent male basketball players. J Strength Cond Res. 2011;25(2):441–452. 20453686 10.1519/JSC.0b013e3181b62be3

[28] Cohen J. Statistical power analysis for the social sciences. 2nd ed. Hillsdale New Jersey: Erlbaum; 1988.

[29] Ho J, Tumkaya T, Aryal S, et al. Moving beyond P values: data analysis with estimation graphics. Nat Methods. 2019;16(7):565–566. 31217592 10.1038/s41592-019-0470-3

[30] Page P. Beyond statistical significance: clinical interpretation of rehabilitation research literature. Int J Sports Phys Ther. 2014;9(5):726–736. 25328834 PMC4197528

[31] Santos EJ, Janeira MA. The effects of resistance training on explosive strength indicators in adolescent basketball players. J Strength Cond Res. 2012;26(10):2641–2647. 22108528 10.1519/JSC.0b013e31823f8dd4

[32] Bedoya AA, Miltenberger MR, Lopez RM. Plyometric training effects on athletic performance in youth soccer athletes: a systematic review. J Strength Cond Res. 2015;29(8):2351–2360. 25756326 10.1519/JSC.0000000000000877

[33] Khlifa R, Aouadi R, Hermassi S, et al. Effects of a plyometric training program with and without added load on jumping ability in basketball players. J Strength Cond Res. 2010;24(11):2955–2961. 20938357 10.1519/JSC.0b013e3181e37fbe

[34] Faigenbaum AD, McFarland JE, Keiper FB, et al. Effects of a short-term plyometric and resistance training program on fitness performance in boys age 12 to 15 years. J Sports Sci Med. 2007;6(4):519–525. 24149486 PMC3794493

[35] Santos EJ, Janeira MA. Effects of complex training on explosive strength in adolescent male basketball players. J Strength Cond Res. 2008;22(3):903–909. 18438223 10.1519/JSC.0b013e31816a59f2

[36] Grindstaff PM, Kreider RB, Bishop R, et al. Effects of creatine supplementation on repetitive sprint performance and body composition in competitive swimmers. Int J Sport Nutr. 1997;7:330–346. 9407259 10.1123/ijsn.7.4.330

[37] Izquierdo M, Ibañez J, Gorostiaga EM. Efectos de la suplementación con creatina sobre la potencia muscular, la resistencia y la velocidad en jugadores de Balonmano. PubliC E Standard. 2006.

[38] Vieira IP, de Paula AG, Gentil P, et al. Effects of creatine supplementation on lower-limb muscle endurance following an acute bout of aerobic exercise in young men. Sports (Basel). 2020;8(2). DOI:10.3390/sports8020012 PMC7077271 31973185

[39] Rosene J, Matthews T, Ryan C, et al. Short and longer-term effects of creatine supplementation on exercise induced muscle damage. J Sports Sci Med. 2009;8(1):89–96. 24150561 PMC3737793

[40] Silva MJCE, Figueiredo A, Carvalho HM, et al. Functional capacities and sport-specific skills of 14- to 15-year-old male basketball players: size and maturity effects. Eur J Sport Sci. 2008;8(5):277–285.

[41] Bogdanis GC, Ziagos V, Anastasiadis M, et al. Effects of two different short-term training programs on the physical and technical abilities of adolescent basketball players. J Sci Med Sport. 2007;10(2):79–88. 16824797 10.1016/j.jsams.2006.05.007

[42] Delextrat A, Martinez A. Small-sided game training improves aerobic capacity and technical skills in basketball players. Int J Sports Med. 2014;35(5):385–391. 24129991 10.1055/s-0033-1349107

[43] Bemben MG, Lamont HS. Creatine supplementation and exercise performance: recent findings. Sports Med. 2005;35(2):107–125. 15707376 10.2165/00007256-200535020-00002

[44] Escribano Ott I, Ibanez Santos J. The role of nutrition in the recovery of a basketball player. Nutr Hosp. 2020;37(1):160–168. 31755278 10.20960/nh.02577

[45] Lyons M, Al-Nakeeb Y, Nevill A. The impact of moderate and high intensity total body fatigue on passing accuracy in expert and novice basketball players. J Sports Sci Med. 2006;5(2):215–227. 24259994 PMC3827563

[46] Yanni SB. Translational ADMET for drug therapy: principles, methods, and pharmaceutical applications. 2015. John Wiley \& Sons.

[47] Petrigna L, Karsten B, Marcolin G, et al. A review of countermovement and squat jump testing methods in the context of public health examination in adolescence: reliability and feasibility of current testing procedures. Front Physiol. 2019;10:1384. 31787902 10.3389/fphys.2019.01384 PMC6853898

